# A forecast social return on investment analysis of a privately operated multi-purpose arena in Japan

**DOI:** 10.3389/fspor.2026.1926992

**Published:** 2026-08-26

**Authors:** Kazuma Aoi, Hiroto Shoji, Hiroaki Ninomiya

**Affiliations:** 1 Graduate School of Health and Sports Science, Doshisha University, Kyoto, Japan; 2 Faculty of Health and Sports Science, Doshisha University, Kyoto, Japan

**Keywords:** arenas, cultural events, forecast analysis, Japan, social value, sport policy, SROI

## Abstract

Japan is expanding arena development, yet the social value of privately financed multi-purpose venues remains under-examined. This study estimates the prospective social value of GLION ARENA KOBE, a privately financed and operated multi-purpose arena in Kobe, Japan, using a forecast Social Return on Investment (SROI) approach, and examines whether projected social value varies by residents' primary purpose of visit. A six-stage forecast SROI framework was applied prior to the arena's opening in April 2025. Data were collected through stakeholder interviews (*n* = 12), a resident willingness-to-pay survey (*n* = 500, of whom 250 were Kobe residents), and secondary financial projections to estimate inputs, outputs, and outcomes; the analysis is based on projected visitor numbers and anticipated outcomes rather than observed post-opening data. Projected annual inputs were ¥5.78 billion (USD 38.2 million) and anticipated outcomes ¥15.31 billion (USD 101.1 million), yielding a forecast SROI ratio of 2.65 in year one. When this total was distributed across residents' primary purposes of visit, concerts were the most frequently cited purpose (39.4% of the ratings recorded), corresponding to ¥6.03 billion of projected social value, followed by everyday park use (¥3.75 billion); event-related purposes together accounted for roughly three-quarters of the distribution, underlining the importance of both cultural programming and freely accessible public space. Sensitivity tests returned forecast SROI ratios of 1.45–3.25 across scenarios varying deadweight (15%–25%), attribution (10%–30%), displacement (5%–15%), projected resident visits (±20%), and the utilization rate (6.8–10.8%). Findings suggest that policy appraisal of arena developments should extend beyond economic multipliers to include outcomes such as civic pride, wellbeing, safety, and education, and that cultural programming alongside sport can enhance social returns where venues integrate quality public space. This study provides the first comprehensive forecast SROI of a privately operated multi-purpose arena in Japan, establishing a baseline for future evaluative SROI analysis; findings should be interpreted as prospective estimates subject to validation once actual operational data become available.

## 1 Introduction

There is increasing interest in evaluating the broader societal impacts of sport infrastructure, with Social Return on Investment (SROI) emerging as a key framework for capturing social value beyond traditional economic impact assessments. SROI helps to visualize intangible social outcomes and provides monetary evidence to support social initiatives (1, 2). Previous research on community sport and leisure facilities has been limited and partial, often valuing only selected social impacts without fully accounting for total operating costs (3). However, multi-purpose arenas are expected to serve not only as venues for sports but also as cultural hubs, attracting large numbers of visitors for concerts, events, and public leisure activities.

In Japan, both public and private investments in arena development are expanding rapidly, creating an urgent need to assess the social value of privately financed and operated arenas. While economic impact studies using a Sport Satellite Account (SSA) have quantified the economic contribution of sport infrastructure, they typically focus on GDP, employment, and tax revenue, leaving broader social outcomes—such as health, wellbeing, civic pride, and community cohesion—unmeasured (4, 5). SROI offers a complementary framework that can capture these wider social benefits and provide a more holistic assessment of arena value.

This study aims to contribute to this emerging field by providing a comprehensive forecast calculation of the social value and SROI of a privately financed and operated multi-purpose arena at the local level. Specifically, it addresses the following two research questions:

1. What social value is projected to be generated by a privately financed and operated multi-purpose arena?
2. How is the projected social value of a multi-purpose arena distributed across residents’ primary purposes of visit—sport, music events, and public leisure?

By applying a forecast SROI methodology to GLION ARENA KOBE prior to its opening, this study provides prospective evidence of social value creation that can inform policy decisions, investment appraisals, and operational strategies for multi-purpose arena development in Japan and internationally. As a forecast study, it also establishes a baseline for future evaluative SROI analysis once actual operational data become available.

## 2 Policy context

### 2.1 International arena and stadium policy

Internationally, arenas and stadiums are positioned as critical infrastructure for advancing both social outcomes and economic growth. In the United Kingdom, the government's Sporting Future strategy (6) established a five-outcome framework emphasizing physical wellbeing, mental wellbeing, individual development, social and community development, and economic development. Recent policy continues to target persistent inactivity while keeping the sport sector accessible and sustainable (6, 7). The UK government is also exploring the development of a Sport Satellite Account (SSA) and a new Economic Snapshot framework to measure the economic contribution of sport through indicators such as GDP, employment, and tax revenue (7). However, these frameworks focus primarily on economic outcomes and do not capture the broader social value dimensions emphasized in Sporting Future.

In the United States, public funding for stadium and arena construction has been controversial, with debates centered on economic impact claims and opportunity costs (8, 9). Studies have shown mixed evidence on the economic benefits of publicly subsidized venues, leading to increased scrutiny of public investment decisions. In response, some jurisdictions have begun requiring social impact assessments alongside economic analyses.

### 2.2 Japanese arena policy and the stadium/arena reform initiative

In Japan, the Japan Sports Agency (JSA), established in October 2015 under the Ministry of Education, Culture, Sports, Science and Technology (MEXT), has positioned multi-purpose arenas and stadiums as key infrastructure for regional revitalization and community development. The JSA's Stadium and Arena Reform Initiative (スタジアム・アリーナ改革), launched under the Second Sport Basic Plan (2017–2021), aims to transform traditional single-purpose sport venues into multi-functional community hubs that generate economic and social value through diverse programming including sports, concerts, cultural events, and public leisure activities (10–12).

The initiative emphasizes three core principles: (1) multi-purpose functionality to maximize utilization and revenue, (2) integration with surrounding urban development to create vibrant public spaces, and (3) sustainable business models that reduce reliance on public subsidies. The JSA has published guidelines encouraging private sector investment and operation of arenas, recognizing that privately operated venues can achieve financial sustainability while delivering public benefits (11).

Despite this policy emphasis, existing economic assessments of Japan's sport sector have focused primarily on macroeconomic metrics—such as Gross Value Added and input–output frameworks—through the development of Sport Satellite Accounts (4, 5), while the broader social outcomes central to the Stadium and Arena Reform Initiative remain underexplored. While these economic assessments are valuable, they do not capture the broader social outcomes—health, wellbeing, civic pride, safety, education, and social capital—that are central to the Stadium and Arena Reform Initiative's vision of arenas as community hubs. This gap between policy aspirations and evaluation practice creates a need for complementary frameworks that can assess social value alongside economic impact (13).

### 2.3 The need for SROI in arena policy evaluation

SROI offers a complementary framework to SSA by monetizing social outcomes that are typically excluded from economic impact assessments. While SSA quantifies the economic footprint of sport infrastructure through market transactions, SROI captures non-market social value through stakeholder consultation and valuation techniques such as willingness-to-pay (WTP) (3, 14). SROI analysis follows seven core principles established by Nicholls et al. (13), including involving stakeholders, understanding what changes, and valuing the things that matter, ensuring transparency and methodological rigor in social value measurement. By integrating SROI with SSA-type economic assessments, policymakers and investors can obtain a more complete picture of arena value that aligns with the holistic objectives of the Stadium/Arena Reform Initiative.

This study applies forecast SROI methodology to a privately operated multi-purpose arena in Japan, providing a structured prospective estimate of social value creation grounded in stakeholder consultation and established financial proxies that can inform investment decisions, policy appraisals, and operational strategies. As Japan continues to expand arena development, this research contributes to the evidence base needed to evaluate whether privately operated multi-purpose venues can deliver the social outcomes envisioned in national sport policy.

## 3 Literature review

### 3.1 The concept of social value

Social value refers to the broader benefits that organizations, projects, or policies create for individuals, communities, and society beyond direct economic returns (13). In the context of sport infrastructure, social value encompasses outcomes such as improved health and wellbeing, enhanced social capital and community cohesion, increased civic pride and place attachment, educational attainment, and perceived safety (15, 16). These outcomes are often intangible and difficult to quantify using traditional economic metrics, yet they are central to the policy rationale for public and private investment in sport facilities.

The concept of social value has gained prominence in public policy and social investment, particularly in the UK where the Public Services (Social Value) Act 2012 requires public authorities to consider social value in procurement decisions. Recent policy frameworks in the UK and Australia have broadened the rationale for public investment in sport, recognizing its contribution not only to economic development but also to wellbeing, social cohesion, and community development (7, 17). This evolution of viewpoints highlights the need for policymakers to consider the multifaceted impact of sport beyond simple economic indicators and to adopt an integrated approach that prioritizes social value alongside economic considerations.

### 3.2 SROI framework

SROI is a principles-based framework for measuring and accounting for social, environmental, and economic value. Developed by the SROI Network, SROI builds on principles of cost-benefit analysis while incorporating stakeholder perspectives and assigning monetary values to outcomes that are not traded in markets (13). As a result, SROI has been proposed as a broader approach to assessing value for money, complementing conventional economic evaluation methods by capturing a wider range of social outcomes and stakeholder impacts (3, 18).

#### 3.2.1 SROI principles

SROI is guided by seven core principles established by Nicholls et al. (13), building on earlier SROI guidelines (19):

1. Involve stakeholders: Stakeholders should inform what is measured and how value is measured.
2. Understand what changes: Articulate how change is created and evaluate this through evidence gathered, recognizing positive and negative changes as well as intended and unintended changes.
3. Value the things that matter: Use financial proxies to recognize the value of outcomes, making decisions about allocating resources between different options.
4. Only include what is material: Determine what information and evidence must be included to give a true and fair picture, such that stakeholders can draw reasonable conclusions about impact.
5. Do not over-claim: Only claim the value that activities are responsible for creating.
6. Be transparent: Demonstrate the basis on which the analysis may be considered accurate and honest, and show that it will be reported to and discussed with stakeholders.
7. Verify the result: Ensure appropriate independent verification of the account.

#### 3.2.2 SROI stages

SROI analysis typically follows six stages as outlined by Nicholls et al. (13):

1. Establishing scope and identifying stakeholders: Define the boundaries of the analysis and identify key stakeholders who experience change as a result of the activity.
2. Mapping outcomes: Develop a theory of change (impact map) that shows the relationship between inputs, activities, outputs, and outcomes for each stakeholder group.
3. Evidencing outcomes and giving them a value: Collect data on outcomes and use financial proxies (e.g., WTP, revealed preference, cost savings) to monetize them.
4. Establishing impact: Adjust outcome values to account for deadweight (what would have happened anyway), attribution (contribution of others), displacement (negative effects elsewhere), and drop-off (decline in outcome over time).
5. Calculating the SROI: Compare the total present value of outcomes to the total value of inputs to calculate the SROI ratio.
6. Reporting, using, and embedding: Share findings with stakeholders, use results to inform decision-making, and embed SROI into organizational processes.

#### 3.2.3 Forecast vs. Evaluative SROI

SROI can be conducted as either a forecast (predictive) or evaluative (retrospective) analysis (13). Forecast SROI is conducted before an activity or project begins, using projected data and assumptions to estimate prospective social value. It is useful for investment appraisal, policy planning, and resource allocation decisions. Evaluative SROI is conducted after an activity has taken place, using actual outcome data to assess realized social value. It is useful for accountability, learning, and demonstrating impact to stakeholders.

Forecast SROI relies on assumptions about future visitor numbers, outcome utilization and visit-frequency parameters, and financial proxy values, making it inherently uncertain. However, it provides valuable prospective evidence for decision-making and establishes a baseline for future evaluative analysis. This study employs a forecast SROI approach, analyzing projected social value before the arena's opening.

### 3.3 SROI in sport: A growing evidence base

The application of SROI to sport contexts has grown significantly in recent years, though it remains less common than traditional economic impact studies. Case studies that employ the SROI framework can be identified at multiple levels of analysis, including national-level investigations, regional-level studies, and project-specific evaluations (2, 16, 20–23).

A systematic review by Gosselin et al. (1) of SROI applications in physical activity and sport interventions identified 17 studies and reported wide variation in SROI ratios across interventions and contexts. The review also highlighted that the large majority (94%) of SROI studies in sport were non-peer-reviewed grey literature, underscoring the importance of rigorous peer-reviewed analysis such as that undertaken in the present study.

At the national level, Davies et al. (16) developed a comprehensive SROI model for sport participation in England, estimating a national SROI ratio of 1.91. Sport England (20) subsequently updated this framework. At the regional level, Hopkinson (21) and ICF GHK (22) demonstrated SROI ratios for community coaching and participation programs. At the project level, Lombardo et al. (23) applied SROI to sport companies in Italy, while Oshimi et al. (2) conducted an SROI analysis of a Japanese professional football club's CSR activities (SROI ratio: 5.3)—providing the most directly comparable precedent in the Japanese context.

In relation to sport facilities, Davies et al. (3) conducted one of the first comprehensive SROI analyses of sport facilities in the UK, measuring 12 community sport and leisure facilities in Sheffield (SROI ratio: 1.20–3.42) and emphasizing total operating cost accounting and impact adjustments. Studies addressing sport facilities otherwise remain scarce, with evidence largely confined to grey literature (24).

Orlowski and Wicker (14) provided a critical review of monetary valuation methods for non-market goods and services in sport, including WTP, contingent valuation, and revealed preference approaches. Their work provides methodological justification for using WTP-based financial proxies in SROI analyses of sport infrastructure.

### 3.4 Research gap and contribution

Despite this growing evidence base, SROI has rarely been applied to large-scale, privately operated multi-purpose arenas. While economic impact studies of arenas are common (8, 9), comprehensive SROI analyses that capture broader social outcomes remain rare, particularly for privately operated multi-purpose venues in Asia (3, 24). No prior study has applied forecast SROI methodology to a privately financed arena in Japan.

This study addresses this gap by:

− Applying forecast SROI methodology to a privately operated arena before its opening;
− Examining how social value varies by the primary purpose of visit; and
− Integrating stakeholder consultation, resident WTP surveys, and secondary data.

Beyond addressing an empirical gap, this study contributes methodologically by demonstrating how forecast SROI can be applied to privately financed sport infrastructure, building on established SROI principles and valuation approaches. It also establishes a baseline for future evaluative SROI analysis by estimating the prospective social value of a multi-purpose arena before actual operational data become available.

## 4 Materials and methods

### 4.1 Research design: forecast SROI

This study employs a forecast SROI approach to estimate the prospective social value of GLION ARENA KOBE prior to its opening in April 2025. As outlined in Section 3.2.3, forecast SROI uses projected data and assumptions to provide prospective evidence for investment appraisal and policy planning, rather than assessing outcomes that have already been realized. This design is appropriate given that the study was conducted before the arena commenced operations, and that no post-opening outcome data were yet available.

The forecast approach has direct implications for how findings should be interpreted. Social value estimates are derived from projected visitor numbers, anticipated outcome utilization and visit-frequency parameters, and stated WTP values collected prior to opening, each of which carries inherent uncertainty. Results should therefore be understood as prospective estimates rather than verified outcomes, and are subject to validation through future evaluative SROI once actual operational data become available (13). This study also serves as a methodological baseline: by documenting assumptions, proxy values, and analytical decisions at the forecast stage, it enables systematic comparison with evaluative findings in subsequent research.

#### 4.1.1 Case study: GLION ARENA KOBE

The choice of GLION ARENA KOBE as the study site reflects both its policy relevance and its research novelty. As a privately financed and operated multi-purpose arena, it represents the type of venue increasingly promoted under the Japan Sports Agency's Stadium/Arena Reform Initiative, yet one for which no SROI evidence existed prior to this study. Its combination of ticketed events and publicly accessible leisure space also allows for purpose-specific analysis of social value, an area that remains underexplored within the sport SROI literature (1).

### 4.2 SROI framework and principles

This analysis follows the six-stage SROI framework established by Nicholls et al. (13), applying each stage sequentially to estimate the projected social value of GLION ARENA KOBE in its first year of operation. The seven SROI principles—outlined in Section 3.2.1—guided analytical decisions throughout, particularly the commitments to stakeholder involvement, avoiding over-claiming, and transparency of assumptions. Each stage is described below. The analysis proceeded through six sequential stages: (Stage 1) establishing scope and identifying stakeholders; (Stage 2) mapping outcomes through a theory of change; (Stage 3) evidencing outcomes and giving them a value; (Stage 4) establishing impact by adjusting for deadweight, attribution, displacement, and drop-off; (Stage 5) calculating the SROI ratio; and (Stage 6) reporting, using, and embedding the findings.

#### 4.2.1 Stage 1: establishing scope and identifying stakeholders

The scope of this analysis is the first year of operation (April 2025–March 2026) of GLION ARENA KOBE, a privately financed and operated multi-purpose arena in Kobe, Japan. The arena is designed to host sports events (basketball, volleyball, ice hockey), concerts, cultural events, and public leisure activities. It features integrated public space including a rooftop park, cafes, and community gathering areas. Stakeholders were identified following the stakeholder-centered approach recommended by Nicholls et al. (13). Four key stakeholder groups were identified: visitors (the local populace attending concerts, professional sports, MICE, and other events, as well as users of the public space); arena operators (the operating company, investors, and sponsors); event hosts (the Kobe Storks professional basketball team and concert and event promoters); and commercial stakeholders and local government (surrounding-area businesses and Kobe City). Of these, visitors—more specifically Kobe residents—were identified through stakeholder consultation as the group for whom the arena generates distinct, non-market social value. Accordingly, while the impact map below documents outcomes for all four groups, only visitor outcomes were monetized in the SROI; the predominantly commercial and economic outcomes accruing to the other three groups are addressed through the operator's separate economic impact assessment and are excluded here to avoid double-counting (13).

Formal stakeholder consultation was limited by the forecast nature of the study and the pre-opening timing. Twelve stakeholder interviews were conducted with arena management (*n* = 4), local government officials (*n* = 3), local business representatives (*n* = 3), and community organization leaders (*n* = 2) between November 2023 and January 2024. These interviews informed the identification of material outcomes and the development of the impact map. The constraints of pre-opening consultation are discussed as a methodological limitation in Section 6.4.

#### 4.2.2 Stage 2: mapping outcomes (theory of change)

An impact map (theory of change) was developed to articulate the causal pathway from inputs and activities to outputs and outcomes for each stakeholder group, as presented in Table 2. The map was informed by stakeholder interviews, established SROI guidance (13), practical applications of theory-of-change mapping in SROI research (2), and prior sport facility studies (3). Material outcomes were selected based on stakeholder input and evidence from prior research on arena impacts.

#### 4.2.3 Stage 3: evidencing outcomes and giving them a value

##### 4.2.3.1 Data collection

Data were collected between November 2023 and January 2024 through three primary sources. Stakeholder interviews (*n* = 12): Semi-structured interviews with arena management, local government officials, local business representatives, and community leaders to identify material outcomes and inform the impact map. Interviews lasted 45–90 min and were conducted in person or via video conference.

Resident WTP survey (*n* = 500): An online survey of residents of Hyogo Prefecture (aged 18–75) was conducted in December 2023 to assess awareness of the arena development, anticipated usage, and WTP for social outcomes. Quota sampling was used to obtain 500 completed responses, stratified by area of residence, gender, and age: 250 respondents resided in Kobe City and 250 in other municipalities of Hyogo Prefecture. The wider prefectural sample was included because the arena's catchment extends beyond the city boundary into the surrounding Hanshin area, which is reflected in the supply-side visitor projection described above. Respondents were asked about their likelihood of visiting the arena, their primary anticipated purpose of visit, their expected visit frequency, their expected expenditure per visit, and their WTP for social outcomes such as civic pride, perceived safety, and community identity. All 500 respondents completed the survey.

Because the outcomes monetized in this study accrue to Kobe residents, the parameters entering the outcome valuation were derived from the 250 Kobe respondents. These comprise the average expected visit frequency of 2.9 visits per year, the expenditure values used to estimate visitor expenditure on the input side, and the unit WTP values reported in Table 3, for which “don't know” responses were treated as missing, leaving 154–158 valid responses per item. Two parameters were instead derived from the full sample of 500 respondents, because they describe expected usage of the park and plaza across the arena's catchment area rather than the valuation of outcomes by Kobe residents: the utilization rate of 8.8% (44 of 500 respondents rating such use as “definitely use”) and the park-specific visit frequency of 4.35 visits per year applied in the supply-side projection of everyday park visits. The purpose-of-visit distribution reported in Table 5 is likewise based on the Kobe respondents, as described in Section 5.5. The sample underlying each parameter is set out in full in Supplementary Table S2.

WTP elicitation followed a payment-card format. For each outcome, respondents selected one of 15 monetary bands ranging from “less than ¥1,000” to “¥60,000 or more” (in ¥5,000 increments above ¥1,000), with a “don't know” option. A payment-card format was chosen in preference to an open-ended format, which imposes a higher cognitive burden and yields more non-responses, and in preference to a dichotomous-choice format, which is sensitive to starting-point bias.

The payment scenario used an equivalent-expenditure frame rather than a tax or admission-fee vehicle. Each question named a single outcome, described how the arena was expected to improve it, and asked the maximum amount the respondent would be willing to pay for other leisure or related expenditure in order to obtain an equivalent improvement. For example, the physical health item read: “If the construction of TOTTEI increases opportunities to watch sport, concerts, and events nearby and to participate in sport locally, and thereby enriches daily life and improves your health, how much would you be willing to pay per month for other leisure expenditure, such as hobbies or dining, to obtain an equivalent improvement in health?” Values were elicited on a monthly basis for physical health and on a per-visit basis for the remaining five outcomes. This frame was selected because a tax vehicle is prone to protest responses in the Japanese context and because a private-expenditure frame remains comparable across ticketed and non-ticketed outcomes. Importantly, no aggregate WTP for the arena as a whole was elicited and subsequently apportioned; each outcome was valued separately, with the target outcome named explicitly, which is how the instrument distinguished individual outcome values from the overall value of the facility.

In converting responses to monetary values, the midpoint of each band was assigned, with the lowest band (“less than ¥1,000”) coded as ¥999 and the highest band top-coded at ¥60,000. Because the payment card is bounded above, the top-coding operates as a conservative treatment of extreme values and no further outlier trimming was applied. “Don't know” responses (92–96 of the 250 Kobe respondents, depending on the item) were treated as missing rather than assigned a value of zero; valid responses therefore numbered 154–158 per item. Mean, median, and interquartile values for each outcome are reported in Table 3, and the full questionnaire is provided in the Supplementary Material.

Secondary data. Projected visitor numbers were estimated in two stages in collaboration with the arena operator. In the first stage, the total number of visitors was estimated separately for each event category (professional basketball games, concerts, and MICE events) by multiplying the number of events in the annual operation plan by the expected attendance per event. Expected attendance figures were derived from publicly available benchmark data: average attendance for B.League games was sourced from B.League official statistics (25); average attendance for concerts was sourced from the annual industry survey of the Concert Promoters Association of Japan (26); average attendance for MICE events was sourced from the Japan National Tourism Organization's international congress statistics (27); and figures for daily use and sightseeing purposes were calculated based on indicators such as visitor numbers for the Kobe Port Tower (28). In the second stage, the share of Kobe City residents among total visitors was estimated for each event category using residential area distribution data from comparable events and facilities. Specifically, the residential distribution of B.League spectators was sourced from a study of Toyama Grouses spectators (29); the residential distribution of concert attendees was sourced from a visitor survey for a large-scale outdoor concert event (30); the residential distribution for MICE events was sourced from visitor surveys for comparable events in Shizuoka City (31); the residential distribution of other event attendees was sourced from a visitor survey for a comparable event (32); and the residential distribution for daily use and sightseeing purposes was estimated based on the operator's projected ratio of visitors between daily use and sightseeing. Financial data, including operational cost projections and revenue forecasts, were provided directly by the arena's operator as part of the collaborative data-sharing arrangement and are not publicly available.

Based on the two-stage estimation described above, total projected visits to the arena in the first year of operation amount to 2,386,229. Of these, an estimated 806,119 visits (33.8%) are by Kobe City residents, distributed across four categories: sports events (100,661; 12%), concerts and cultural events (100,012; 12%), other events including MICE (34,400; 4%), and public park and plaza everyday users (571,046; 71%). The relatively high share of everyday park and plaza users reflects the arena's design as an integrated urban precinct incorporating publicly accessible outdoor spaces, and this segment was estimated separately from ticketed events using comparable facility usage data. Table 1 sets out the derivation by event category, showing for each category the number of event days, the expected attendance per event, the projected number of visits, the assumed share of Kobe residents, and the resulting number of resident visits.

**Table 1:** Projected visits by event category and estimated Kobe-resident visits (first year of operation).

Event category	Event days per year	Expected attendance per event	Projected visits	Kobe-resident share	Kobe-resident visits
Basketball (B.League)	30	6,711	201,323	50%	100,661
Concerts (international artists)	15.6	9,146	142,678	15%	21,402
Concerts (domestic artists)	57.3	9,146	524,066	15%	78,610
MICE and corporate events	12	9,000	108,000	20%	21,600
Other events	8	4,000	32,000	40%	12,800
Everyday park use and sightseeing	365	—	1,378,162	41.4%	571,046
Total	—	—	2,386,229	33.8%	806,119

Event days and expected attendance per event were compiled from the operator's annual operation plan and the benchmark sources described in Stage 3. Kobe-resident shares were derived from residential distribution data for comparable events and facilities. Everyday park use was estimated separately from ticketed events: the Kobe-resident component (571,046 visits) is the product of the city population (1,492,017), the utilization rate (8.8%), and the park-specific stated visit frequency (4.35 visits per year), both derived from the full survey sample; the remainder comprises visitors from the wider Hanshin area and sightseeing visitors. Figures are rounded and may not sum exactly. A flowchart summarizing the relationship between this supply-side projection and the population-based basis used for outcome valuation is provided in the Supplementary Material (Figure S1).

##### 4.2.3.2 Quantifying outcomes (utilization and visit frequency)

Rather than applying a single incidence rate to the total number of visitors, outcomes were quantified using utilization and visit-frequency parameters based on the resident population.

For each outcome, the annual per-capita value derived from the WTP survey was multiplied by the population of Kobe City as of December 2024 (1,492,017 (33);) and by a conservative utilization rate of 8.8%, representing the proportion of survey respondents who indicated that they would “definitely use” the public park and plaza (44 of the 500 respondents; see the sampling description in Stage 3).

For outcomes valued on a per-visit basis, the per-visit WTP value was converted into an annual per-capita value using the average expected visit frequency of 2.9 visits per year, as reported by Kobe residents in the questionnaire survey. For physical health, which was valued on a monthly basis, the monthly value was multiplied by 12 to derive an annual per-capita value.

Accordingly, this study adopted a population-based estimation approach rather than an event attendance-based approach, reflecting its objective of measuring the social value accruing to local residents through both attendance at arena events and everyday use of publicly accessible spaces such as the park and plaza.

Both parameters were benchmarked against external data and are conservative relative to those benchmarks. Municipal surveys of park use in Japan report substantially higher actual usage than the 8.8% applied here: in Yokohama City, 53.8% of surveyed residents reported using urban parks at least monthly and 26.3% at least weekly (34), and the national Urban Park Use Survey similarly documents frequent repeat use (35). Counting as users only those respondents who indicated they would “definitely use” the park and plaza (44 of 500) therefore yields a utilization rate well below observed usage rates for comparable public spaces.

For visit frequency, national data indicate that 26.2% of Japanese adults attended a sport event in person at least once in the preceding year (36); the corresponding revealed rate among the Kobe respondents in this study was 23.6%, which supports the representativeness of the sample. The same respondents reported a mean of 4.7 in-person spectating days per year, including non-attenders, which exceeds the 2.9 visits per year applied in the valuation. Kobe respondents who rated park use as “strongly applicable” stated an intended frequency of 13.3 visits per year, again substantially above the value applied. Two distinct frequency parameters are used in the analysis and are reported separately for transparency: the projection of everyday park and plaza visits on the supply side applies the park-specific stated frequency of 4.35 visits per year (the full-sample mean for that item), whereas the valuation of outcomes applies the lower cross-category mean of 2.9 visits per year. Because the stated intentions underlying both parameters were collected before opening, the potential divergence between stated intentions and revealed behaviour is discussed in Section 6.4.

##### 4.2.3.3 Financial proxies (WTP values)

Financial proxies were used to monetize outcomes following the approach recommended by Orlowski and Wicker (14). As Japan-specific WTP values were unavailable in the existing literature for many of the outcomes examined, values were elicited directly through the resident survey, using the responses of the 250 Kobe residents included in the sample. The outcome framework and survey design were informed by Davies et al. (3), an SROI analysis of community sport and leisure facilities in the United Kingdom that monetized comparable outcomes (including health and wellbeing). The selection of outcome categories was further informed by social value estimation frameworks developed by EY Strategy and Consulting (37) and the Development Bank of Japan (38), which identify relevant domains of social value generated by sport facilities and events in Japan.

For reporting, yen values are also expressed in US dollars using the 2024 average exchange rate of USD 1 = ¥151.37 (39). This survey-based valuation approach is consistent with forecast SROI practice, where the primary objective is prospective estimation rather than precise valuation (14). Nevertheless, the reliance on stated WTP values collected prior to the arena's opening remains a limitation of the study. This issue is discussed in Section 6.4, and sensitivity analyses examining the robustness of the results under alternative assumptions are presented in Section 5.4.

#### 4.2.4 Stage 4: establishing impact (adjusting for deadweight, attribution, displacement, and drop-off)

To avoid over-claiming, outcome values were adjusted for deadweight, attribution, displacement, and drop-off.

Deadweight was estimated using the observed city-wide growth in visitation as a proxy for the counterfactual trend, and was set at 18.4% in the base case. According to municipal tourism statistics, total visitor arrivals in Kobe City rose from 21.61 million in 2022 to 26.45 million in 2023, a year-on-year increase of 22.4% associated with the post-pandemic recovery of tourism demand (28). The increment corresponds to 18.3% of current-year arrivals ((26.45–21.61)/26.45), and deadweight was set at 18.4%, marginally above this trend-derived value. A city-level proxy was necessary because facility-specific counterfactual data cannot be observed prior to opening, and neither expert elicitation nor a Delphi procedure was undertaken. Two features of this proxy should be noted. First, it is drawn from visitation rather than from the outcomes themselves, and therefore approximates rather than directly measures the share of outcomes that would have arisen without the arena. Second, because the single-year recovery rate is unusually high by historical standards, the proxy is likely to overstate the underlying counterfactual trend, so that 18.4% represents a conservative, outcome-reducing assumption. Given this uncertainty, deadweight is varied between 15% and 25% in the sensitivity analysis reported in Section 5.4.

By contrast, attribution and displacement were set at 0% in the base case. This reflects the forecast nature of the present study, which was conducted prior to the arena opening. At the time the stakeholder interviews were conducted, the arena had not yet been constructed, making it difficult to assess reliably the contribution of external organizations to the anticipated outcomes (attribution) or the extent to which the arena would displace existing facilities or services (displacement). Accordingly, reliable empirical estimates of these parameters could not be obtained from a resident survey conducted prior to the arena opening. Rather than adopting speculative assumptions, attribution and displacement were therefore held at 0% in the base case.

It should be acknowledged explicitly that holding attribution and displacement at 0% is not a neutral choice: to the extent that other organizations contribute to the same outcomes, or that activity is drawn from existing venues without generating additional social value, this assumption biases the base-case ratio upward and therefore risks over-claiming. The direction of this bias is examined quantitatively in Section 5.4, where scenarios raising both parameters simultaneously are reported.

Recognizing the uncertainty inherent in forecast SROI and the risk of over-claiming, sensitivity analyses were subsequently undertaken using alternative assumptions for attribution (10%–30%) and displacement (5%–10%), while maintaining deadweight at 18.4%.

Drop-off was not applied because the SROI ratio reported in this study relates only to the first year of operation. A five-year present value projection, reported as a supplementary analysis in the Supplementary Material (Table S1), applies an annual discount rate to subsequent years. The discount rate was adopted from the methodology developed by the Development Bank of Japan (38).

To operationalize these impact adjustments, adjusted outcome values were calculated using Equation 1:

AdjustedOutcomeValue=(OutcomeValue)×(1−Deadweight)×(1−Attribution)×(1−Displacement) (1)

As drop-off was not applicable in the first year of operation, it was excluded from the calculation.

#### 4.2.5 Stage 5: calculating the SROI

The SROI ratio was calculated as:

SROI=TotalPresentValueofOutcomes/TotalValueofInputs (2)

Inputs comprised visitor expenditure during the first year of operation, together with the arena's operating costs and capital costs. Outcomes were aggregated across all stakeholder outcome categories based on the impact map developed in Stage 2. In addition to the base-case SROI ratio, sensitivity analyses were conducted to examine the robustness of the results under alternative assumptions.

## 5 Results

### 5.1 Impact map (theory of change)

Table 2 presents the impact map for GLION ARENA KOBE, articulating the causal pathway from inputs and activities to outputs and outcomes for each stakeholder group. The map was developed through stakeholder interviews and informed by prior SROI studies in sport and policy frameworks from the Japan Sports Agency's Stadium/Arena Reform Initiative.

**Table 2:** Impact map (theory of change) for GLION ARENA KOBE.

Stakeholder Group	Inputs	Activities	Outputs	Outcomes
Visitors (local populace for concerts, pro-sports, MICE, other events)	¥3.33 billion (USD 22.0 m): Spending on admission fees, transport, food and beverages, merchandise, accommodation and tourism	Attending events and using the venue and its park	Number of visits to the arena	Health(physical health, wellbeing), individual development (educational attainment, civic pride), social development (social capital, perceived safety)
Arena operators (operator, investors and sponsors)	¥1.24 billion (USD 8.2 m): Construction and operational costs	Driving community-rooted arena operations; naming rights, sponsorship, and sponsor-led event planning	Establishment as a hub and iconic destination for the sports and entertainment industry in the Kansai region; increased corporate awareness; improved brand image; improved employee engagement	Synergy with group and affiliated companies; strengthened brand power and corporate image
Event hosts (Kobe Storks, promoters, etc.)	¥1.21 billion (USD 8.0 m): venue rental income	Hosting matches, concerts, and events	Increased spectator numbers at an arena people want to attend; greater motivation among concert and event attendees; greater motivation among athletes wishing to join the team	Improved business momentum through the arena; strengthened competitiveness of the team
Commercial stakeholders and local government (surrounding-area businesses, One Bright KOBE sponsors, Kobe City)	Not monetized: promotional support, stakeholder introductions, and administrative time	Commercial stakeholders: event publicity and PR for the arena opening. Local government: responding to MICE demand, connecting local businesses, and raising event awareness	Creating new MICE demand; increase in people interested in culture and sport; enhanced attractiveness of Kobe as a city	Commercial stakeholders: increased visitor exchange and circulation across the waterfront and Meriken Park areas. Local government: greater area recognition for Shinminatomachi

The impact map documents anticipated outcomes for all four stakeholder groups. However, only the social outcomes accruing to visitors (health, individual development, and social development) were monetized in the SROI analysis. The outcomes anticipated for arena operators, event hosts, and commercial and government stakeholders are predominantly commercial and economic in nature and are assessed through the operator's separate economic impact analysis; they are therefore excluded from the SROI valuation to avoid double-counting (13).

### 5.2 Inputs

Total inputs for the first year of operation were ¥5.78 billion (USD 38.2 million), aggregated across stakeholder groups rather than by cost type (Table 2).

The largest share (57.7%), amounting to ¥3.33 billion (USD 22.0 million), comprised visitor expenditure. This value was estimated by calculating the average expenditure for each visit purpose—including professional sports events, concerts, other events, and everyday use—from the questionnaire survey and multiplying these values by the projected total number of Kobe City resident visits for each visit purpose, as estimated using the methodology described in Stage 3. Visitors using the arena for non-event purposes were assumed not to incur admission fees.

The arena operator's input comprised construction and operational costs, accounting for ¥1.24 billion (USD 8.2 million). Construction costs were annualized over the expected facility lifespan to provide a conservative estimate consistent with the SROI principle of not over-claiming (13).

Event host inputs comprised projected venue rental revenue associated with the planned program of sports events, concerts, and other events, totalling ¥1.21 billion (USD 8.0 million).

Commercial stakeholders in the surrounding area and local government provided promotional support, stakeholder introductions, and administrative time. Although these inputs required real resources, stakeholder interviews concluded that they were not substantial enough to warrant monetary valuation and were therefore recorded as non-monetized.

Construction and operational costs were estimated with reference to publicly available financial data from comparable domestic arenas, and were subsequently validated through stakeholder interviews with the arena operator to ensure the plausibility of the estimates.

**Figure 1:**
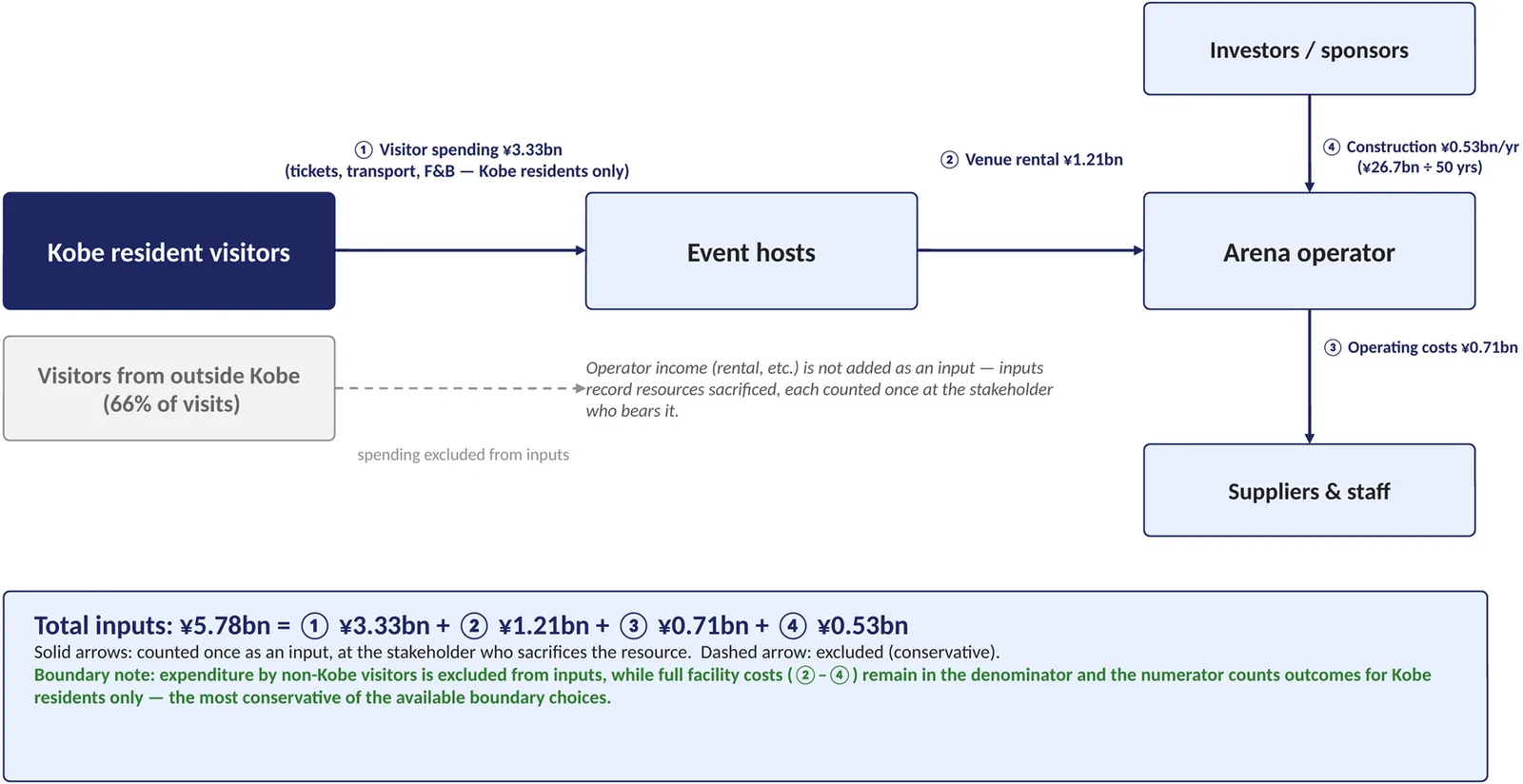
Financial flows among stakeholders and the scope of inputs (first year of operation). Solid arrows denote resource flows recorded once as an input at the stakeholder that sacrifices the resource; the dashed arrow denotes expenditure excluded from the analysis.

Because the same financial resources could in principle circulate between stakeholders—for example, ticket expenditure by visitors may pass through event hosts and subsequently finance venue rental paid to the operator—the boundary applied to inputs is set out explicitly in Figure 1. Inputs comprise four mutually exclusive items, each recorded once and attributed to the stakeholder that sacrifices the resource: visitor expenditure by Kobe residents; venue rental paid by event hosts; operating costs borne by the operator; and construction costs annualized over the expected facility lifespan. Revenue received by the operator is not additionally recorded as an input, so no yen is counted on both the expenditure and the receipt side. Venue rental is financed from event revenues generated by attendees nationwide, of which expenditure by Kobe residents forms only a part; moreover, any residual overlap between these items would inflate the denominator, so that its removal would raise rather than lower the ratio. Retaining both items is therefore the conservative treatment, and is consistent with the practice in prior facility-level SROI studies of recording visitor expenditure alongside operating costs (3).

The asymmetry between the numerator and the denominator warrants explicit justification. Outcomes are monetized for Kobe residents only, whereas the denominator carries the full construction and operating costs of the facility. At the same time, expenditure by visitors from outside Kobe—who account for approximately two-thirds of projected visits—is excluded from inputs, consistent with the exclusion of their outcomes from the numerator. Including non-resident outcomes together with their expenditure would raise the ratio, since the associated outcome values would exceed the additional expenditure recorded. The boundary adopted here is therefore the most conservative of the available options, and follows the SROI principle of not over-claiming (13).

### 5.3 Outcomes and financial proxies

Table 3 presents the outcomes included in the analysis, their unit WTP values, and the method by which annual values were calculated. Outcomes were selected on the basis of three criteria: stakeholder materiality, as identified through interviews; evidence from prior SROI studies of sport facilities (1–3); and alignment with the Japan Sports Agency's Stadium/Arena Reform Initiative. Economic development outcomes (employment, GDP contribution) were excluded to avoid double-counting with separate economic impact assessments; environmental outcomes and crime reduction were not identified as material by stakeholders and fall outside the scope of this analysis.

**Table 3:** Summary of outcomes and WTP valuation.

Outcome Category	Unit WTP, mean (¥)	Median (¥)	Interquartile range (¥)	Valid n	Calculation Method 1	Calculation Method 2
Health: Physical health	¥5,547/month	¥3,000	¥999–¥7,500	158	Monthly WTP × 12	Outcome × population (1,492,017) × usage rate (8.8%)
Health: Wellbeing	¥6,365/visit	¥3,000	¥999–¥7,500	156	WTP × 2.9 visits	Outcome × population (1,492,017) × usage rate (8.8%)
Individual development: Educational attainment	¥4,844/visit	¥3,000	¥999–¥7,500	154	WTP × 2.9 visits	Outcome × population (1,492,017) × usage rate (8.8%)
Individual development: Civic pride	¥4,832/visit	¥3,000	¥999–¥7,500	158	WTP × 2.9 visits	Outcome × population (1,492,017) × usage rate (8.8%)
Social development: Social capital	¥5,103/visit	¥3,000	¥999–¥7,500	154	WTP × 2.9 visits	Outcome × population (1,492,017) × usage rate (8.8%)
Social development: Perceived safety	¥5,295/visit	¥3,000	¥999–¥4,125	154	WTP × 2.9 visits	Outcome × population (1,492,017) × usage rate (8.8%)

Total Projected Outcomes (Base Case, after 18.4% deadweight): ¥15.31 billion (USD 101.1 million).

WTP values were obtained from the 250 Kobe residents included in the resident survey (*n* = 500, December 2023) and are reported per month or per visit as indicated. Annual totals were calculated using the methodology described in Section 4.2. USD values were converted using the 2024 average exchange rate of USD 1 = ¥151.37 (39). Values were elicited using a payment card with 15 monetary bands; band midpoints were assigned, with the lowest band coded as ¥999 and the highest top-coded at ¥60,000. “Don't know” responses were treated as missing, giving 154–158 valid responses per item out of 250 Kobe respondents.

Although the impact map (Table 2) identifies outcomes for all four stakeholder groups, only the social outcomes accruing to visitors—spanning health, individual development, and social development—were monetized and included in the SROI calculation. The outcomes anticipated for arena operators, event hosts, and commercial and government stakeholders (for example, strengthened brand value, improved business momentum, and wider visitor circulation) are predominantly commercial and economic in nature. These were therefore treated as part of the venue's economic impact, which is assessed separately by the operator, and were excluded from the SROI valuation to avoid double-counting and to remain consistent with the principle of not over-claiming (13). Restricting monetization to visitor outcomes also reflects the materiality judgement, reached through stakeholder consultation, that local residents are the group for whom the arena generates distinct, non-market social value.

### 5.4 SROI calculation and sensitivity analysis

In the base case, outcome values incorporate a deadweight deduction of 18.4% and a 3.5% annual discount rate, with attribution and displacement set at 0% (see below). On this basis, the first-year forecast SROI ratio is:

Applying Equation 2,

SROI=¥15.31billion/¥5.78billion=2.65

This indicates that, under the projected assumptions, every ¥1 invested in the arena is associated with ¥2.65 of projected social value during the first year of operation. Because a forecast SROI rests on assumptions that cannot be verified before opening, sensitivity analyses were conducted across five parameters: deadweight (15%–25%), attribution (10%–30%), displacement (5%–15%), the projected number of resident visits (±20%, which affects visitor expenditure on the input side), and the utilization rate (6.8–10.8%, which affects the outcome side). Table 4 reports the resulting ratios.

**Table 4:** Sensitivity analysis: SROI ratios under alternative scenarios.

Scenario	Deadweight	Attribution	Displacement	Other parameter varied	SROI
Base case	18.4%	0%	0%	—	2.65
Lower impact adjustment	18.4%	10%	5%	—	2.27
Higher impact adjustment	18.4%	30%	10%	—	1.67
Stringent impact adjustment	18.4%	30%	15%	—	1.58
Lower deadweight	15%	0%	0%	—	2.76
Higher deadweight	25%	0%	0%	—	2.44
Most stringent combination	25%	30%	15%	—	1.45
Resident visits −20%	18.4%	0%	0%	Projected resident visits −20%	3.00
Resident visits +20%	18.4%	0%	0%	Projected resident visits +20%	2.38
Lower utilization rate	18.4%	0%	0%	Utilization rate 6.8%	2.05
Higher utilization rate	18.4%	0%	0%	Utilization rate 10.8%	3.25

All scenarios apply a 3.5% annual discount rate. Variation in projected resident visits affects visitor expenditure in the denominator; variation in the utilization rate affects projected outcomes in the numerator. Ranges for attribution and displacement follow Davies et al. (3).

The projected SROI remains above 1.0 in every scenario tested. Under the stricter scenario recommended in the SROI literature for forecast studies, in which attribution and displacement are raised simultaneously to 30% and 15%, the ratio is 1.58; under the most stringent combination tested, which additionally raises deadweight to 25%, it is 1.45. Varying the projected number of resident visits by ±20% yields ratios of 2.38–3.00: because outcomes are estimated from the resident population rather than from attendance, a reduction in projected visits lowers visitor expenditure in the denominator without altering the numerator, and the ratio consequently rises. Varying the utilization rate by ±2 percentage points, which acts directly on the numerator, yields ratios of 2.05–3.25. Taken together, these results indicate that the central finding—that projected social value exceeds projected inputs—is robust to substantial variation in the key assumptions, including to any residual double counting among outcome categories discussed in Section 6.4.

### 5.5 Comparison of outcomes by purpose of visit

To address Research Question 2, the total projected social value of ¥15.31 billion was allocated across residents' primary purposes of visit in proportion to the ratings of “strongly applicable” given by Kobe respondents to each purpose (94 such ratings from 59 respondents; Table 5). Because the purpose item was administered as a set of separate rating scales rather than as a single forced choice, a respondent could rate more than one purpose as strongly applicable; the resulting shares therefore describe the distribution of stated purposes across ratings rather than a partition of respondents. Because WTP, visit frequency, and outcome incidence were not estimated separately for each purpose, this analysis describes how the projected social value is distributed across primary purposes of visit; it does not establish that any purpose intrinsically generates more social value per resident or per visit.

**Table 5:** Projected outcomes by purpose of visit (base case).

Primary Purpose of Visit	Share of Ratings (%)	Total Outcome Value (¥, USD)
Concerts	39.4%	¥6.03 billion (USD 39.8 m)
Park use	24.5%	¥3.75 billion (USD 24.8 m)
Watching sport	20.2%	¥3.09 billion (USD 20.4 m)
Other events	16.0%	¥2.44 billion (USD 16.1 m)
Total	100%	¥15.31 billion (USD 101.1 m)

Values are obtained by allocating the total projected social value of ¥15.31 billion in proportion to the ratings of “strongly applicable” given by Kobe respondents to each purpose as their primary anticipated purpose of visit (94 ratings from 59 respondents; a respondent could rate more than one purpose as strongly applicable). WTP, visit frequency, and outcome incidence were not estimated separately by purpose; the figures therefore describe the distribution of the aggregate projection rather than purpose-specific estimates of value.

Among the “strongly applicable” ratings given by Kobe respondents, attending concerts was the most frequently cited purpose of visit (39.4% of ratings), followed by park use (24.5%), watching sport (20.2%), and attending other events (16.0%).

**Figure 2:**
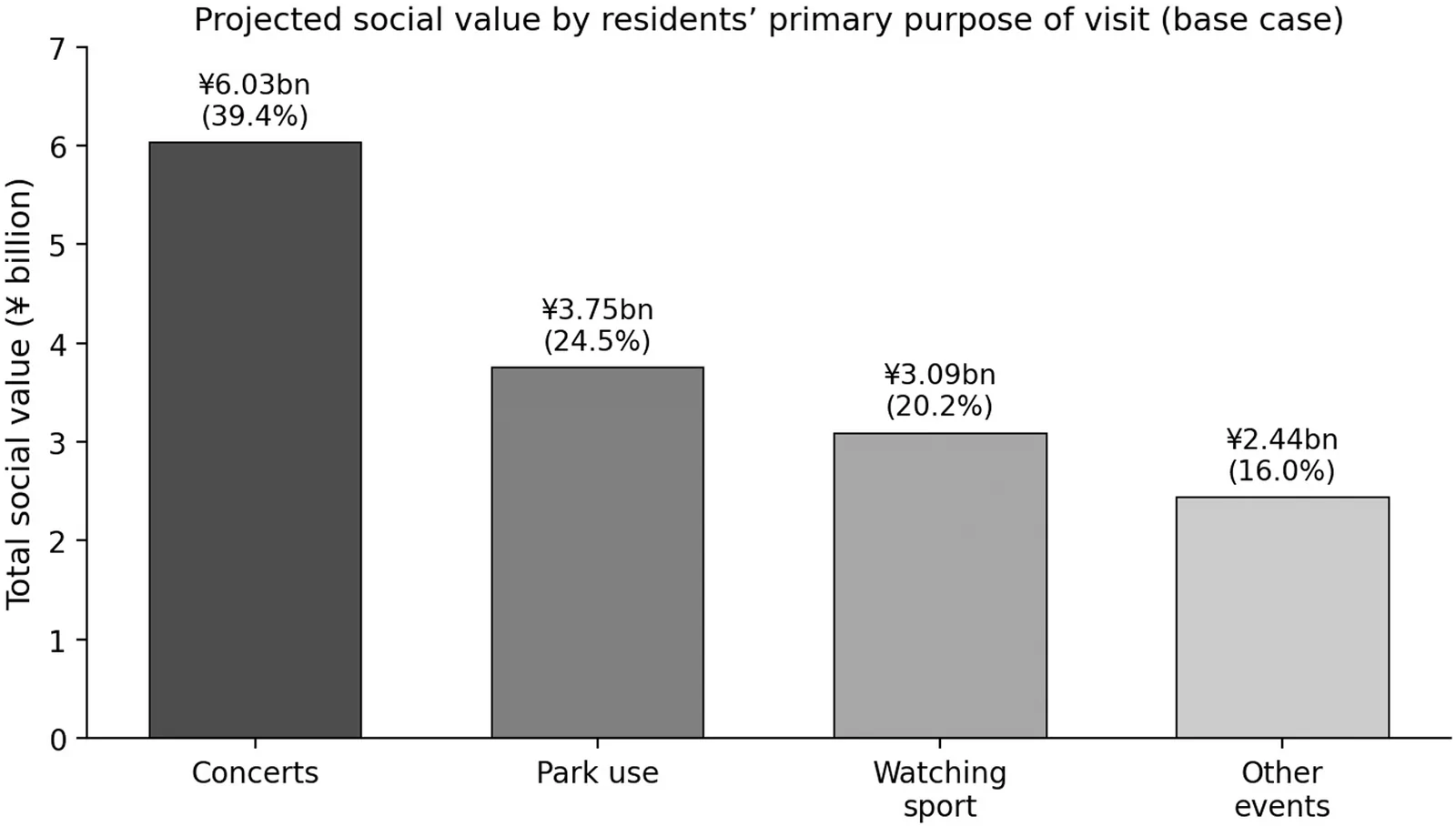
Projected social value by residents’ primary purpose of visit (base case). Percentages indicate the share of the “strongly applicable” ratings recorded for each purpose as a primary reason for visiting.

Correspondingly, of the total projected social value of ¥15.31 billion generated during the first year of operation, concerts accounted for the largest share at ¥6.03 billion (USD 39.8 million), followed by park use at ¥3.75 billion (USD 24.8 million), watching sport at ¥3.09 billion (USD 20.4 million), and other events at ¥2.44 billion (USD 16.1 million) (Figure 2).

Taken together, the share of the projected social value distribution associated with residents whose primary purpose is attending concerts, sport, or other programmed events amounted to ¥11.57 billion (USD 76.4 million), representing 75.5% of the total. This indicates that, although approximately one quarter of the distribution is associated with the publicly accessible park, the majority is associated with residents whose primary motivation for visiting is event attendance.

## 6 Discussion

### 6.1 Interpretation of findings

This forecast SROI analysis projects that GLION ARENA KOBE would generate ¥2.65 of social value for every ¥1 invested in the first year of operation, with total projected outcomes of ¥15.31 billion against projected inputs of ¥5.78 billion. This ratio falls within the range reported in prior SROI studies of sport facilities and programs. It sits within the range of 1.20–3.42 reported by Davies et al. (3) for twelve community sport and leisure facilities in Sheffield, and above the ratio of 1.91 estimated for sport participation in England (16). It is below the ratio of 5.3 reported by Oshimi et al. (2) for the corporate social responsibility activities of a Japanese professional soccer team, plausibly because the present study monetizes outcomes for Kobe residents alone while retaining the full facility costs in the denominator, whereas programme-level analyses typically carry narrower cost bases. Unlike these evaluative studies, the present analysis is prospective, and its contribution lies in demonstrating the feasibility of forecast SROI for appraisal prior to investment commitment; its principal weakness, correspondingly, is its reliance on stated intentions and projected attendance, as discussed in Section 6.4. Taken together, these comparisons suggest that privately operated multi-purpose arenas may be capable of generating substantial social value alongside economic returns.

Several structural features of the arena contribute to its projected social value. First, multi-purpose programming across sports, concerts, cultural events, and public leisure attracts visitor segments with distinct outcome profiles, thereby maximizing aggregate social value.

Second, the integration of a publicly accessible park for everyday use generates substantial social value through outcomes related to health, wellbeing, and perceived safety, particularly for users who do not attend ticketed events. The projected social value associated with this publicly accessible space accounts for approximately one quarter of the total.

Third, the arena is projected to enhance civic pride and place attachment for both visitors and non-visiting residents, contributing to community identity and urban regeneration in the Kobe waterfront area.

Fourth, physical health and wellbeing outcomes represent the largest outcome categories in absolute value terms, driven by sport participation, everyday park use, and cultural engagement.

### 6.2 Variation by purpose of visit

The disaggregated analysis describes how the projected social value is distributed across residents' primary purposes of visit.

The largest share of the distribution is associated with concert attendance (¥6.03 billion; 39.4% of ratings), reflecting the prevalence of concerts as residents' primary anticipated purpose of visit. This suggests that cultural programming is a major channel through which residents expect to engage with the arena, and is therefore central to its social contribution rather than merely complementing sport. It does not, however, imply that concerts intrinsically generate more social value per visit than sport attendance, since the underlying valuation parameters were not estimated separately by purpose.

The second largest share of the distribution is associated with everyday park use, accounting for ¥3.75 billion (USD 24.8 million), accounting for 24.5% of ratings. This finding is particularly noteworthy because the value in question arises through freely accessible, non-ticketed public space rather than organized events. It is also consistent with the Japan Sports Agency's Stadium/Arena Reform Initiative, which emphasizes the development of multi-functional community hubs, highlighting the importance of integrating publicly accessible spaces into arena design.

Watching sport (¥3.09 billion; 20.2%) and attending other events (¥2.44 billion; 16.0%) accounted for the remaining share, broadly consistent with previous research on the social value of sport facilities (3).

These findings collectively suggest that operators seeking to maximize social value should pursue balanced programming across concerts, sport, and other events, reflecting the breadth of purposes that residents anticipate, and that the provision of publicly accessible space, even where it generates no ticket revenue, is associated with a substantial share of the projected social value distribution (approximately one quarter).

### 6.3 Policy implications

This study carries several implications for arena policy and investment appraisal. First, SROI provides a framework that complements existing economic tools such as Sport Satellite Accounts and economic impact assessments. While these instruments capture GDP contribution, employment, and tax revenue, they do not quantify social outcomes such as health, wellbeing, civic pride, or social capital. Integrating SROI into appraisal processes would enable more holistic valuation of arena investments and support evidence-based resource allocation.

Second, the findings provide empirical support for the objectives of the Japan Sports Agency's Stadium/Arena Reform Initiative. The projected social outcomes—health promotion, community development, and urban regeneration—align directly with the Initiative's emphasis on multi-purpose, community-oriented facilities. Forecast SROI, as demonstrated here, can serve as an appraisal tool for proposed developments ahead of investment commitment.

Third, the substantial projected social value associated with park use—around a quarter of the total, arising from freely accessible non-ticketed space—strengthens the case for mandating or incentivizing quality public space within arena development approvals. Policymakers should consider whether planning frameworks adequately account for the social value of freely accessible, non-ticketed space.

Fourth, the finding that the largest share of the projected social value distribution is associated with concert attendance suggests that operators should resist narrowing their program mix to high-margin ticketed sport events alone. Balanced scheduling across sport and cultural events maximizes social returns in ways that a sport-only model would not.

### 6.4 Limitations and future research

As a forecast SROI, this study is subject to several important limitations that should inform interpretation and direct future research.

Visitor numbers, outcome utilization and visit-frequency parameters, and event schedules are based on projections rather than observed data, and actual outcomes may diverge from these estimates due to market conditions, programming decisions, or unforeseen disruptions. Future evaluative SROI using actual operational data is therefore essential to validate the findings.

Stakeholder consultation was constrained by the pre-opening timing. The study relied on a resident survey (*n* = 500, including 250 Kobe residents) and twelve stakeholder interviews. The interview sample was small and, in composition, weighted towards supply-side and institutional perspectives—arena management, local government, local business, and community organization leaders—with no direct participation by ordinary residents. Although the resident survey captures residents' anticipated use and valuations, the identification of material outcomes and the materiality judgement may therefore under-represent the diversity of residents' views across age groups, income brackets, and districts. Future evaluative SROI should incorporate focus groups with residents at the theory-of-change stage, alongside post-visit surveys of actual arena users. Testing alternative weightings across stakeholder groups would also be informative; because monetized outcomes in the present study accrue to a single group, such weighting does not enter the quantitative model here, but it becomes applicable once outcomes for additional stakeholder groups are monetized.

Impact adjustment estimates, particularly attribution and displacement, were based on stakeholder consultation and assumptions rather than empirical observation. Although sensitivity analysis tested a range of conservative scenarios, actual adjustments may differ once the arena is operational. Future evaluative SROI should use observed operational data, and where possible, control groups or quasi-experimental designs to estimate these factors more rigorously.

The utilization rate and visit frequency were derived from intentions stated before the arena opened. Although both parameters are conservative relative to the external benchmarks reported in Section 4.2, a divergence between stated intentions and revealed post-opening behaviour cannot be excluded, and future evaluative SROI should replace these parameters with observed attendance and usage data.

The aggregation of WTP values across the six outcomes also warrants caution. Each item was elicited separately using an equivalent-expenditure frame that named the outcome explicitly, and no aggregate facility-level value was apportioned. Nevertheless, respondents may not have fully separated conceptually adjacent outcomes such as wellbeing, civic pride, and social capital, and the summed values may therefore capture overlapping benefits to some degree. To the extent that such overlap exists, the numerator is overstated. The stricter scenarios reported in Section 5.4, under which the ratio remains above 1.0, bound the influence of this risk, and the treatment of “don't know” responses as missing rather than zero is a further source of upward bias in the mean WTP values that should be borne in mind when interpreting the estimates.

Finally, the comparison by purpose of visit allocates the total projected social value in proportion to residents' stated primary purposes. Because outcome incidence, WTP, and visit frequency were not estimated separately by purpose, the purpose-level figures should be read as a distribution of the aggregate projection rather than as purpose-specific estimates of value. Estimating purpose-specific outcome models represents a further avenue for future research.

Finally, the exclusion of economic outcomes means that the SROI ratio of 2.65 reflects social value alone. A comprehensive assessment integrating SROI with economic impact analysis would capture total value more fully and represents an important direction for future research.

## 7 Conclusion

This study provides the first comprehensive forecast SROI analysis of a privately operated multi-purpose arena in Japan, projecting that GLION ARENA KOBE would generate ¥2.65 of social value for every ¥1 invested in the first year of operation.

The findings suggest that multi-purpose programming combining sport, concerts, and everyday park use could generate substantial prospective social value through outcomes related to physical health, wellbeing, civic pride, social capital, educational attainment, and perceived safety, with the distribution of that value across residents' primary purposes of visit highlighting distinct channels of engagement. Concert attendance, the most frequently cited primary purpose, is associated with the largest single share of the projected social value distribution (¥6.03 billion), while everyday park use is associated with approximately one quarter of the total (¥3.75 billion) through freely accessible public space. These findings underscore the importance of both balanced programming and the integration of publicly accessible spaces in the design and operation of multi-purpose arenas.

As a forecast SROI, the findings should be interpreted as prospective estimates rather than verified outcomes, and require validation through future evaluative analysis using actual operational data. Key limitations—including the reliance on projected visitor numbers and utilization parameters, the pre-opening timing of stakeholder consultation, and uncertainty surrounding impact adjustments—are inherent to the forecast approach and reinforce the need for subsequent evaluative SROI.

This study contributes to the growing evidence base on SROI in sport by extending the methodology to a privately operated multi-purpose arena in Japan and demonstrating how forecast SROI can complement conventional economic impact assessments in policy appraisal. By making social value visible and directly comparable with economic value, SROI provides a more holistic framework for evaluating arena developments, consistent with the objectives of the Japan Sports Agency's Stadium/Arena Reform Initiative and broader international sport policy frameworks that emphasize wellbeing and community development.

Future research should undertake evaluative SROI using actual operational data, compare projected and realized social value, refine forecast methodologies, and strengthen the evidence base regarding the long-term social value generated by multi-purpose arenas.

## Data Availability

The raw data supporting the conclusions of this article will be made available by the authors, without undue reservation.
