# Supplementary material for: A forecast social return on investment analysis of a privately operated multi-purpose arena in Japan
Source: Front Sports Act Living. 2026 Aug 26;8:1926992. doi: 10.3389/fspor.2026.1926992 (PMC13553851; doi:10.3389/fspor.2026.1926992)
Supplement: Supplementary file 1 [file Supplementaryfile1.docx]

**Supplementary Material**

*A Forecast SROI Analysis of a Privately Operated Multi-Purpose Arena in Japan*

This Supplementary Material provides (S1) a flowchart reconciling the supply-side visitor projection with the population-based basis used for outcome valuation; (S2) the five-year present value projection of outcomes, reported as a supplementary analysis; and (S3) the willingness-to-pay (WTP) questionnaire items used in the resident survey, presented in the original Japanese with English translations.

**Supplementary Figure S1**

Figure S1. Visitor estimation framework: relationship between the supply-side projection of visits and the population-based basis used for outcome valuation (first year of operation). The supply-side projection determines venue activity and visitor expenditure recorded as inputs. Outcome valuation is derived independently from the resident survey, and assumes 379,157 annual visits, equivalent to 47% of the 806,119 projected Kobe-resident visits.

**Supplementary Table S1**

Table S1. Five-Year Present Value of Outcomes (Base Case, Supplementary Projection)

| **Outcome category** | **Deadwei-ght** | **Attribut-ion** | **Displac-ement** | **Discount** | **Year 1 (¥, USD)** | **Year 2** | **Year 3** | **Year 4** | **Year 5** |
| --- | --- | --- | --- | --- | --- | --- | --- | --- | --- |
| **Health: Physical health** | 18.4% | 0% | 0% | 3.5% | ¥7.13bn (USD 47.1m) | ¥6.88bn (USD 45.5m) | ¥6.64bn (USD 43.9m) | ¥6.41bn (USD 42.3m) | ¥6.18bn (USD 40.8m) |
| **Health: Wellbeing** | 18.4% | 0% | 0% | 3.5% | ¥1.97bn (USD 13.0m) | ¥1.90bn (USD 12.6m) | ¥1.83bn (USD 12.1m) | ¥1.77bn (USD 11.7m) | ¥1.71bn (USD 11.3m) |
| **Individual development: Educational attainment** | 18.4% | 0% | 0% | 3.5% | ¥1.50bn (USD 9.9m) | ¥1.45bn (USD 9.6m) | ¥1.40bn (USD 9.2m) | ¥1.35bn (USD 8.9m) | ¥1.30bn (USD 8.6m) |
| **Individual development: Civic pride** | 18.4% | 0% | 0% | 3.5% | ¥1.49bn (USD 9.8m) | ¥1.44bn (USD 9.5m) | ¥1.39bn (USD 9.2m) | ¥1.34bn (USD 8.9m) | ¥1.30bn (USD 8.6m) |
| **Social development: Social capital** | 18.4% | 0% | 0% | 3.5% | ¥1.58bn (USD 10.4m) | ¥1.52bn (USD 10.0m) | ¥1.47bn (USD 9.7m) | ¥1.42bn (USD 9.4m) | ¥1.37bn (USD 9.1m) |
| **Social development: Perceived safety** | 18.4% | 0% | 0% | 3.5% | ¥1.64bn (USD 10.8m) | ¥1.58bn (USD 10.4m) | ¥1.53bn (USD 10.1m) | ¥1.47bn (USD 9.7m) | ¥1.42bn (USD 9.4m) |
| **Total** |  |  |  |  | **¥15.31bn (USD 101.1m)** | **¥14.78bn (USD 97.6m)** | **¥14.26bn (USD 94.2m)** | **¥13.76bn (USD 90.9m)** | **¥13.28bn (USD 87.7m)** |

Notes: Values are present values of outcomes after applying a deadweight deduction of 18.4% and an annual discount rate of 3.5%, with attribution and displacement set at 0% as in the base case. Year 1 corresponds to the base-case outcome value used in the SROI calculation reported in the main text. This projection is reported as a supplementary analysis only: corresponding five-year inputs, drop-off assumptions, and a cumulative five-year SROI ratio were not estimated, and no cumulative ratio should be inferred from these figures. The SROI ratio reported in the main text relates to the first year of operation. USD values were converted using the 2024 average exchange rate of USD 1 = ¥151.37 (International Monetary Fund, 2024).

**Supplementary Text S1: Willingness-to-Pay Questionnaire Items**

The survey was administered online in Japanese to 500 residents of Hyogo Prefecture (250 of whom resided in Kobe City) in December 2023. Items are reproduced below in the original Japanese with English translations. At the time of the survey the development was referred to by its precinct name, TOTTEI; GLION ARENA KOBE is the arena within that precinct.

**1. Response scale for the WTP items (Q5–Q10)**

All six WTP items used an identical payment card. Respondents selected one of the following options:

| **Code** | **Japanese** | **English translation** |
| --- | --- | --- |
| 1 | １，０００円未満 | Less than ¥1,000 |
| 2 | １，０００円から５，０００円未満 | ¥1,000 to less than ¥5,000 |
| 3–13 | ５，０００円から６０，０００円未満（５，０００円刻み） | ¥5,000 to less than ¥60,000 (in ¥5,000 bands) |
| 14 | ６０，０００円以上 | ¥60,000 or more |
| 15 | わからない | Don't know |

Values were assigned as band midpoints, with code 1 coded as ¥999 and code 14 top-coded at ¥60,000. Code 15 was treated as missing. Q5 was elicited on a monthly basis; Q6–Q10 were elicited on a per-visit basis.

**2. WTP items**

**Q5. Physical health (monthly)**

あなたは、ＴＯＴＴＥＩが建設されることで、現地でのスポーツ観戦やコンサート・イベントなどの娯楽機会や現地でのスポーツ参加機会が増加・充実し、身近に感じられるようになり、生活の充実感が増し、健康度が増すのではないかと考えています。同じように健康度を高めるために、趣味や食事など他の娯楽に支出する場合、月額いくらまでなら支払ってもよいと思いますか？

*You may expect that the construction of TOTTEI will increase and enrich opportunities to watch sport, concerts, and events locally and to participate in sport locally, making them feel closer at hand, enriching daily life, and improving your health. If you were to spend on other leisure activities, such as hobbies or dining, in order to obtain a comparable improvement in health, how much would you be willing to pay per month?*

**Q6. Social capital (per visit)**

…生活の充実感が増し、社会的な繋がり等が増すのではないかと考えています。同じように社会的な繋がり等を高めるために、趣味や食事など他の娯楽に支出する場合、いくらまでなら支払ってもよいと思いますか？

*…enriching daily life and increasing social connections. If you were to spend on other leisure activities, such as hobbies or dining, in order to obtain a comparable increase in social connections, how much would you be willing to pay?*

**Q7. Civic pride (per visit)**

…生活の充実感が増し、地域への愛着や誇りが増すのではないかと考えています。同じように地域への愛着や誇りを高めるために、趣味や食事など他の娯楽に支出する場合、いくらまでなら支払ってもよいと思いますか？

*…enriching daily life and increasing your attachment to and pride in the local area. If you were to spend on other leisure activities in order to obtain a comparable increase in attachment and pride, how much would you be willing to pay?*

**Q8. Educational attainment (per visit)**

…生活の充実感が増し、教育の充実度の増加につながるのではないかと考えています。同じように教育の充実度を高めるために、何らか他に支出する場合、いくらまでなら支払ってもよいと思いますか？

*…enriching daily life and enhancing educational enrichment. If you were to spend on something else in order to obtain a comparable enhancement in educational enrichment, how much would you be willing to pay?*

**Q9. Perceived safety (per visit)**

…生活の充実感が増し、安全性が増すのではないかと考えています。同じように安全性を高めるために、防犯対策など他に支出する場合、いくらまでなら支払ってもよいと思いますか？

*…enriching daily life and increasing safety. If you were to spend on other measures, such as crime prevention, in order to obtain a comparable increase in safety, how much would you be willing to pay?*

**Q10. Wellbeing (per visit)**

…生活の充実感が増し、幸福度が増すのではないかと考えています。同じように幸福度を高めるために、趣味や食事など他の娯楽に支出する場合、いくらまでなら支払ってもよいと思いますか？

*…enriching daily life and increasing your sense of wellbeing. If you were to spend on other leisure activities, such as hobbies or dining, in order to obtain a comparable increase in wellbeing, how much would you be willing to pay?*

Note: Q6–Q10 share the identical stem used in Q5 up to the point marked "…"; only the outcome named and the reference expenditure differ. The full stem is reproduced in Q5.

**3. Related items used in the analysis**

**Q1_1–Q1_5**

あなたがＴＯＴＴＥＩおよびＧＬＩＯＮ　ＡＲＥＮＡ　ＫＯＢＥを利用する場合、想定できる一番の利用目的をお答えください。（スポーツ観戦／コンサート鑑賞／スポーツ・コンサート以外のイベント参加／公園利用／その他）

*What would be your primary anticipated purpose for using TOTTEI and GLION ARENA KOBE? (watching sport / attending concerts / attending events other than sport or concerts / using the park / other). Five-point scale from "strongly applicable" to "not at all applicable". Responses of "strongly applicable" were used to derive the utilization rate (8.8%) and the distribution by primary purpose of visit (Table 5).*

**Q20_1–Q20_5**

あなたは、ＴＯＴＴＥＩが建設された場合、以下の項目においてどのくらいの頻度で参加（または来訪）したいかをお答えください。（週に１回程度／月に１回程度／年に数回程度／年に１回程度／行かない／わからない）

*If TOTTEI were built, how frequently would you wish to participate in or visit each of the following? (about weekly / about monthly / several times a year / once a year / would not go / don't know). Responses were annualized (48, 12, 6, 1, 0 visits per year respectively) to derive the average visit frequency of 2.9 visits per year across categories, and the park-specific frequency of 4.35 visits per year used in the supply-side projection.*

**Q21–Q24**

上でお答えいただいた項目では、イベント開催地域でいくらぐらいお金を使っていますか？チケット代などイベント関連の消費金額、さらに現地での交通費や飲食なども含み、ご自身の平均的な消費金額をお答えください。

*For each purpose, how much do you typically spend in the area where the event is held, including tickets, local transport, and food and beverages? Responses used the same 15-band payment card and were used to estimate visitor expenditure recorded as an input.*

**Supplementary Table S2**

Table S2. Survey flow and the sample used for each parameter

| **Parameter** | **Sample used** | **n** | **Where applied** |
| --- | --- | --- | --- |
| **Total survey sample** | Residents of Hyogo Prefecture, aged 18–75, quota sampling by area, gender and age | 500 (250 Kobe City; 250 other Hyogo municipalities) | — |
| **Utilization rate (8.8%)** | Full sample | 44 of 500 rating park and plaza use as "definitely use" | Number of unique Kobe users (Section 4.2) |
| **Park-specific visit frequency (4.35 visits/year)** | Full sample | 335 valid responses of 500 | Supply-side projection of everyday park visits (Table 1) |
| **Average visit frequency (2.9 visits/year)** | Kobe residents | 250 | Annualization of per-visit WTP values (Section 4.2) |
| **Unit WTP values** | Kobe residents, excluding "don’t know" responses | 154–158 valid per item, of 250 | Outcome valuation (Table 3) |
| **Distribution by primary purpose of visit** | Kobe residents rating a purpose as "strongly applicable" | 94 ratings from 59 respondents, of 250 | Allocation of total value by purpose (Table 5) |
| **Expenditure per visit** | Kobe residents | 250 | Visitor expenditure recorded as an input (Section 5.2) |

Notes: Parameters describing expected usage across the arena’s catchment area were derived from the full sample, whereas parameters entering the monetary valuation of outcomes were derived from the 250 Kobe respondents, consistent with the restriction of monetized outcomes to Kobe residents. Because the purpose item was administered as a set of separate rating scales rather than a single forced choice, a respondent could rate more than one purpose as "strongly applicable".
